# Comparative Genomic Analysis Reveals Distribution, Organization, and Evolution of Ferrous and Sulfur Oxidation Genes in the Genus *Acidithiobacillus*

**DOI:** 10.3390/ijms27156728

**Published:** 2026-07-28

**Authors:** Zulaikha Abid, Yuandong Liu

**Affiliations:** 1 School of Minerals Processing and Bioengineering, Central South University, Changsha 410083, China; 245618003@csu.edu.cn; 2 Key Laboratory of Biohydrometallurgy of Ministry of Education, Changsha 410083, China

**Keywords:** *Acidithiobacillus*, comparative genomics, iron oxidation, sulfur oxidation, bioleaching, acidophiles, synteny, phylogenomics, rusticyanin, metabolic evolution, bioinformatics

## Abstract

*Acidithiobacillus* spp. are key players in metal mobilization and sulfur cycling in acidic environments, with important applications in biomining and bioleaching operations. But the evolutionary organization of the iron and sulfur oxidation systems of these organisms is not well understood. In this study, a comparative genomic analysis of 31 *Acidithiobacillus* genomes (95,180 predicted proteins) was performed to investigate the distribution, conservation, and genomic organization of genes involved in ferrous iron oxidation, reduced sulfur compound oxidation, respiratory electron transfer, and sulfur trafficking. This study identified key metabolic components including *cyc2*, *rus*, *cyc1*, *petA-F*, *coxA-D*, *soxA-D*, *sqr*, *tetH*, *doxA*, *doxD*, *hdrA-C*, *tusA*, and *dsrE-like* genes using genome annotation, conserved-domain identification, orthology inference, and gene-neighborhood analyses. The results indicate that energy metabolism in *Acidithiobacillus* is organized into discrete functional modules rather than as a single universally conserved pathway. The *cox* terminal oxidase complex and the *cyc2/rus/cyc1* iron-entry modules were identified in 21 genomes (67.7%) and 19 genomes (61.3%), respectively. Sulfur associated systems displayed greater variation in gene content, *Sox* genes were found in 10 genomes (32.3%), *SQR/TetH/Dox* components in 12 genomes (38.7%), and the *Hdr/Tus/DsrE* sulfur-transfer system in 22 genomes (71.0%). Phylogenomic analysis revealed iron-enriched, sulfur-enriched and mixed metabolic profiles, in line with differential retention, loss and reorganization of redox modules. These results show that metabolic potential cannot be accurately predicted from single marker genes alone, but must integrate orthology, genomic context and phylogenomic relationships. This comparative genomic framework may facilitate the identification of candidate strains for biomining applications and provide insights into the potential ecological functions of *Acidithiobacillus* species in acidic environments.

## 1. Introduction

Acidophilic chemolithotrophic bacteria are involved in important processes of metal mobilization and sulfur cycling in acidic environments and contribute to natural acid mine drainage systems and industrial biomining operations [1,2]. Among these microorganisms, members of the genus *Acidithiobacillus* are particularly interesting because they gain energy from the oxidation of ferrous iron and reduced inorganic sulfur compounds in very acidic environments [3,4,5,6]. These oxidation reactions produce ferric iron and sulfuric acid, which enhance the dissolution of sulfide minerals and metal recovery in bioleaching processes [4,6,7].

The bacterium *Acidithiobacillus* is highly metabolically versatile; certain species specialize in iron oxidation, and some in sulfur oxidation, with many strains being capable of using both substrates under the appropriate conditions [7,8]. This diversity renders the genus an excellent model system to study microbial adaptation to acidic mineral-rich environments [9]. But the genetic basis for this metabolic variation is relatively poorly understood, particularly regarding the distribution, organization, and evolutionary history of the multiple gene systems involved in iron and sulfur oxidation [3,5]. In *A. ferrooxidans*, this process is mediated by outer membrane and periplasmic cytochromes, rusticyanin, the *bc1 complex*, *iron oxidase (Iro)*, *HiPIP,* and terminal oxidases, and so on [1,3,10,11,12,13,14,15]. This study identified key marker genes (*cyc2*, *rus, cyc1*, *petA-F*, *coxA-D*) that are not always present as a conserved block; their copy number, organization, and distribution are quite different [11]. Thus, iron oxidation is best described as a multi-component electron-transfer system rather than a single-gene trait [5,10].

Sulfur oxidation is even more complicated. Reduced inorganic sulfur compounds can be metabolized by several enzymatic pathways involving thiosulfate, tetrathionate, sulfide, elemental sulfur, and sulfur-transfer intermediates [7]. Genes encoding the Sox system, sulfide oxidoreductase, tetrathionate hydrolase, *Dox/TQO-like* proteins, heterodisulfide reductase-like systems, *TusA*, and *DsrE-like* sulfur-transfer proteins have all been implicated in sulfur metabolism in acidophilic bacteria [16]. Recent reviews of sulfur metabolism in *Acidithiobacillus* highlight that sulfur oxidation is not explained by a single universal pathway and that a number of partially overlapping systems likely operate in different species or environmental settings [14,17,18].

Recent advances in comparative genomics have greatly improved our understanding of the evolution of *Acidithiobacillia.* Acidic environment adaptation has been shaped by gene gain, loss, duplication, and horizontal transfer [15,19,20]. Genome-scale studies have redefined the phylogenetic structure of this class and identified deep phylogenomic divisions. These findings have direct implications for energy metabolism, as iron- and sulfur-oxidation genes might also exhibit lineage-specific patterns of retention, reduction, and reorganization [13]. Furthermore, studies of intraspecific divergence in *A. ferrooxidans* suggest that differences in iron-oxidation proteins such as *rusticyanin* can be involved in strain-level divergence and adaptation to the environment [12,21].

However, a complete comparison of iron- and sulfur-oxidation systems among *Acidithiobacillus* genomes is lacking. Most studies have been undertaken on single species, individual pathways, or the wider evolution of the *Acidithiobacillia* [22,23,24,25]. Only a few have combined gene presence–absence profiling, validation of conserved domains, orthology-based annotation, and gene-neighborhood analysis in a single analytical framework [26]. This integration is important because annotation names can be misleading, especially for broad redox proteins such as *cytochromes*, *oxidoreductases*, *Dox-like* proteins, and sulfur carrier proteins [27].

The analysis of gene neighborhoods is especially useful for pathway conservation. Detection of a marker gene does not necessarily mean that a complete functional pathway is present; co-localization of pathway genes in conserved order supported by local genomic context provides stronger evidence [28]. Another important layer of evidence is validation at the protein level. Public genome annotations differ enormously in product descriptions, gene names, and annotation depth. Conserved-domain resources, orthology-based annotation, and orthogroup inference reduce the likelihood of overinterpretation of broad product labels as pathway-specific genes [4].

A protein labeled “*cytochrome*” or “*oxidoreductase*” may not be part of the desired iron or sulfur oxidation module if its domain architecture, orthology, and genomic context do not support that function. Thus, a robust pathway reconstruction should include gene detection with protein domain evidence, orthology, and local organization [29]. In this study, the genomes of 31 *Acidithiobacillus* strains were analyzed to reconstruct the distribution, organization, and evolutionary diversification of genes involved in ferrous iron oxidation, sulfur oxidation, and respiratory electron transfer [30]. The 95,180 predicted proteins were used for marker detection, conserved-domain validation, orthology-supported annotation, and orthogroup inference [31]. The curated marker genes were grouped into six functional modules, including *cyc2/rus/cyc1*, *petABC/bc1*, *coxABCD*, *Sox*, *SQR/TetH/Dox,* and *Hdr/Tus/DsrE* [32]. These modules were used for comparison of iron-entry, respiratory electron-transfer, terminal oxidase, sulfur-oxidation, and sulfur-transfer systems within the genome set [33].

The primary objective of this work was to find out whether iron and sulfur oxidation systems in *Acidithiobacillus* are conserved as coherent pathways or dispersed as modular, lineage-associated, and locally reorganized genomic systems [34]. The analysis included gene-content profiling and module completeness scoring, conserved-domain and orthology validation, orthogroup support, gene-neighborhood reconstruction, and phylogenomic mapping. This design enabled the interpretation of pathway conservation using multiple layers of evidence [35]. This research provides a genome-wide perspective on Iron and Sulfur oxidation in *Acidithiobacillus* and supports a modular model for metabolic diversification [36]. The analysis clarifies the distribution of iron-enriched, sulfur-enriched, and mixed genomic profiles across the genus by comparing marker distribution, module conservation, and locus architecture [37]. These results are relevant not only for understanding the evolution of acidophilic energy metabolism, but also for selection and interpretation of strains used in biomining and bioleaching systems [16].

## 2. Results

### 2.1. Comparative Genome Analysis

The comparative dataset consisted of 31 *Acidithiobacillus* genomes, representing a significant portion of the species and strain diversity of the genus (Supplementary Table S1). It provides a complete listing of the genome accession numbers, species assignment, strain designations, genome assembly characteristics, and genome quality metrics. After genome quality assessment, eight representative genomes with a variety of iron-rich, sulfur-rich, and mixed metabolic profiles were selected for detailed gene neighborhood, synteny, and comparative genomic analyses (Table 1). These genomes were selected to be representative of the diversity of energy metabolism, but not so numerous as to require detailed structural comparison.

**Figure 1:**
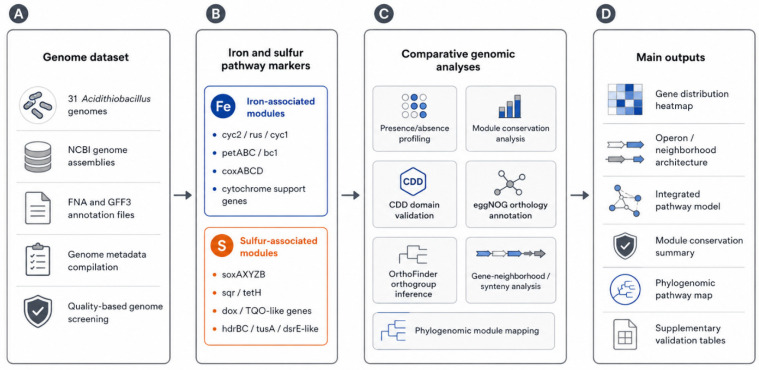
Strategy for comparative genomics to reconstruct iron- and sulfur-oxidation systems in *Acidithiobacillus*. The workflow consists of four stages: (**A**) collection and annotation of the genome, (**B**) finding metabolic markers for iron (blue) and sulfur (orange), (**C**) comparative genomic analysis (gene-content profiling, validation of conserved-domains, gene orthology inference, gene-neighborhood reconstruction, module conservation analysis, and phylogenomic mapping), (**D**) generating the core study outputs. Iron associated modules are indicated by blue boxes, sulfur associated modules are indicated by orange boxes and general analytical procedure and study output boxes are indicated by grey boxes. The sequence of the work steps is shown by arrows.

Candidate pathway-associated proteins were identified using curated marker genes and functional annotations, and were validated by conserved-domain analysis, orthology assignment, and genomic context evaluation. Proteins involved in iron and sulfur metabolism, especially *cytochrome-associated*, *Dox/TQO-like*, and sulfur-transfer proteins, are often misannotated in public databases, requiring multi-step validation. A comparative genomics framework was designed that combined three complementary lines of evidence: (i) gene-content variation, (ii) local pathway organization, and (iii) phylogenomic distribution of functional modules (Figure 1). This approach reduced the reliance on single gene annotations and allowed reconstruction of pathways based on integrated evidence.

### 2.2. Genome-Wide Distribution of Iron- and Sulfur-Oxidation Genes

**Figure 2:**
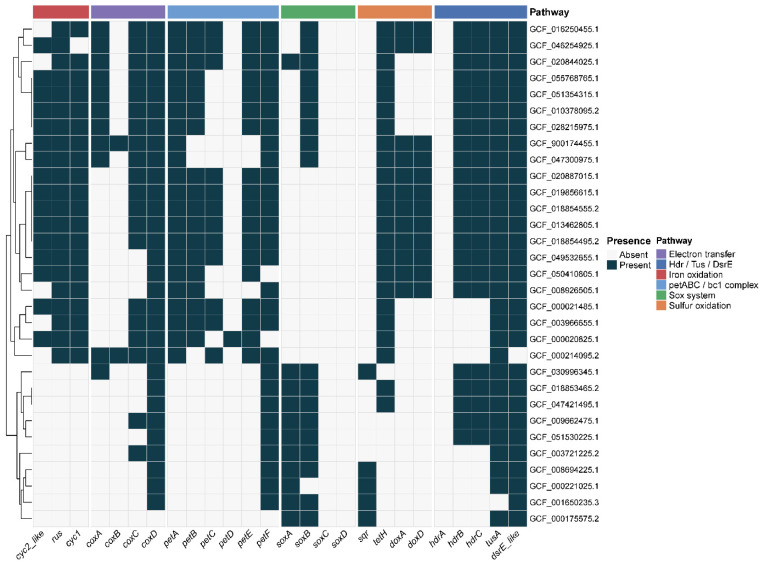
Genome-wide presence–absence profile of curated iron- and sulfur-associated marker genes across 31 *Acidithiobacillus* genomes. Rows are genomes and columns are pathway marker genes. The marker genes were grouped into six functional categories (indicated by the colored bar), including iron uptake, respiratory electron transfer, *terminal oxidases*, *petABC/bc1 complex*, *Sox* system, and *SQR/TetH/Dox/Hdr/Tus/DsrE* sulfur-associated systems. Shaded cells represent gene presence; unshaded cells represent gene absence. Genomes were ordered by hierarchical clustering of similarity in gene presence–absence profiles to highlight common genomic patterns, while marker genes were shown according to their functional classification.

The presence/absence matrix showed substantial variation in the distribution of iron- and sulfur-related genes among the 31 *Acidithiobacillus* genomes (Figure 2). No distinctive pattern of pathway presence was identified among all genomes, indicating that the genus does not rely on a single shared chemolithotrophic strategy. Instead, the distribution of pathway markers broke down genomes into iron-enriched, sulfur-enriched, mixed iron-sulfur, and reduced metabolic profiles.

Genes involved in iron metabolism were *cyc2-like* genes, *rus*, *cyc1*, *petA-F,* and *coxA-D*. Functions for iron uptake and periplasmic electron transfer were located in the *cyc2/rus/cyc1* region, and respiratory electron transfer and terminal oxidase functions were encoded by *petABC/bc1* and *coxABCD*, respectively. Genes involved in sulfur metabolism were *soxA-D*, *sqr*, *tetH*, *doxA*, *doxD*, *hdrA-C*, *tusA,* and *dsrE-like* genes, associated with *Sox*-mediated sulfur oxidation, sulfide/tetrathionate oxidation pathways, and sulfur transfer systems mediated by *Hdr/Tus/DsrE*.

Iron-associated genes were not always co-retained with complete sulfur-associated systems, as shown by the gene-content matrix. Similarly, complete iron-entry modules were not always present in sulfur-rich genomes. These observations are consistent with a modular model of energy metabolism in *Acidithiobacillus* in which iron and sulfur oxidation pathways can be independently maintained, reduced, or rearranged. Also, the heatmap revealed a unique cluster of genomes with high recovery of *cyc2/rus/cyc1, petABC/bc1*, and *coxABCD* modules, distinct from genomes with lower iron-associated systems. Genes related to sulfur showed more variation and different combinations of *Sox*, *SQR/TetH/Dox,* and *Hdr/Tus/DsrE* modules across the dataset.

### 2.3. Partial Conservation and Lineage-Specific Variation of Iron-Oxidation Modules

Iron-related markers were relatively conserved, but not universal. Moreover, 21 out of 31 genomes (67.7%) contained the iron entry and rusticyanin-linked electron transfer system, suggesting that the *cyc2/rus/cyc1* iron-entry module is common but not ubiquitous (Table 1). The iron-entry module is specifically used throughout this work to refer to the combination of *cyc2/rus/cyc1* genes involved in ferrous iron oxidation and electron transfer from iron to the respiratory chain. In contrast, the iron oxidation pathway is used in a broader sense to refer to the entire iron-dependent energy-conserving pathway, including the iron-entry (*cyc2/rus/cyc1*) and electron transfer to *coxABCD* modules. Thus, these terms are not used as synonyms. The *petABC/bc1* respiratory electron-transfer module was encoded in 19 genomes (61.3%), and its distribution was generally similar to the iron-entry module. However, some genomes contained respiratory electron-transfer components without upstream iron-entry genes. The *coxABCD* terminal oxidase module was present in 21 genomes (67.7%) with a similar pattern of distribution.

These results suggest that iron oxidation is better described as a multi-component electron transfer network rather than a single diagnostic pathway. Genomes enriched in iron were considered those containing all three modules (*cyc2/rus/cyc1*, *petABC/bc1*, and *coxABCD*). Genomes with only subsets were given partial or reduced iron-oxidation profiles. Interestingly, the *petABC/bc1* module was sometimes found in genomes that lacked *cyc2/rus/cyc1*, suggesting that components of respiratory electron transfer may have roles in addition to iron oxidation, potentially in sulfur-based energy conservation.

### 2.4. Higher Functional Diversity of Sulfur-Oxidation and Sulfur-Transfer Modules

Sulfur modules displayed greater variation in gene content and module recovery across the analyzed genomes (Table 1). The *Sox* module was detected in only 10 (32.3%) genomes, suggesting *sox*-dependent sulfur oxidation is limited to a subset of lineages, and likely a reflection of ecological specialization or lineage-specific adaptation. The *SQR/TetH/Dox* module was present in 12 genomes (38.7%) and contained genes for *sulfide oxidation (sqr)*, *tetrathionate metabolism (tetH)*, *and Dox/TQO-like* sulfur oxidation (*doxA*, *doxD*, *doxX*). The identification of this enzyme in both *Sox*-positive and *Sox*-negative genomes indicates the existence of multiple pathways for sulfur oxidation.

The *Hdr/Tus/DsrE* sulfur-transfer module was the most widely conserved sulfur-associated module, identified in 22 genomes (71.0%). This broad distribution implies that sulfur transfer and intermediate processing are key to sulfur metabolism in many species of *Acidithiobacillus*. The high conservation reflects that intracellular sulfur trafficking is more evolutionarily stable than specific sulfur oxidation pathways. Unlike the *Sox* or *SQR/TetH/Dox* systems, the inference is that the *Hdr/Tus/DsrE* module is mainly involved in intracellular sulfur transfer and processing, rather than in oxidation of reduced sulfur compounds. Sulfur metabolism overall appeared highly modular, with several overlapping systems that probably favor adaptation to different sulfur substrates in acid environments.

### 2.5. Module-Level Scoring Differentiates Iron-Enriched, Sulfur-Enriched, and Mixed Genome Profiles

**Figure 3:**
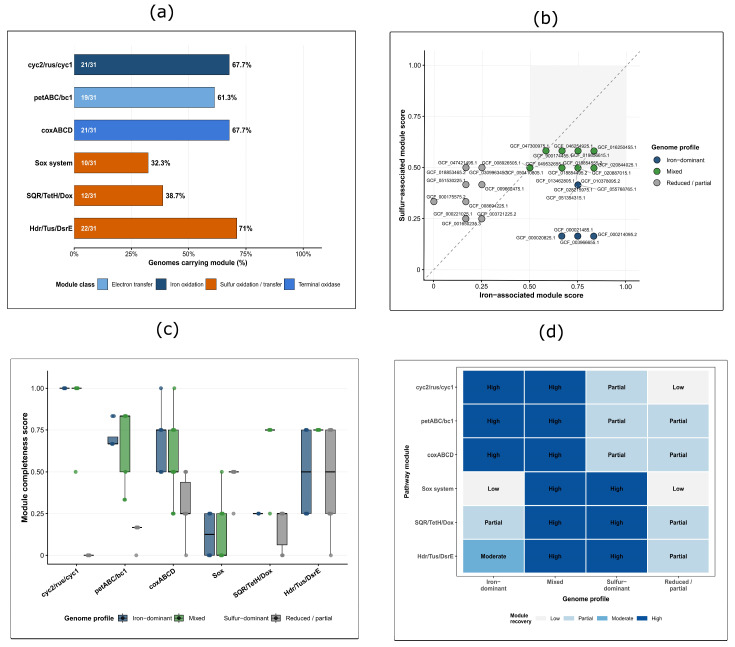
Conservation of pathway modules and genome-level iron–sulfur metabolic profiles across 31 *Acidithiobacillus* genomes. (**a**) Frequency of recovery of the six curated pathway modules across all genomes, expressed as the percentage of genomes containing each module. (**b**) Distribution of genomes according to normalized iron-associated and sulfur-associated module scores, showing the classification of iron-enriched, sulfur-enriched, and mixed metabolic profiles. The dashed diagonal indicates equal iron and sulfur module scores. (**c**) Distribution of module completeness scores for each genome-profile category. Boxes represent the interquartile range, the horizontal line indicates the median, whiskers represent the data range, and points denote individual genomes. (**d**) Summary heatmap showing the relative recovery of each pathway module across the four genome-profile categories (iron-enriched, mixed, sulfur-enriched, and reduced/partial), where darker shading indicates higher module recovery.

To obtain genome-wide metabolic profiles from the presence–absence pattern of each gene, the completeness of the module was evaluated (Figure 3b,c). A proportion of the marker genes detected in each genome was used to calculate the iron module score (IMS) and sulfur module score (SMS) for the genome. A functional module was considered to have at least half of the marker genes in it. This proportional criterion increased the likelihood of not seeing a conserved pathway when the gene was isolated and allowed for the detection of partially retained modules. This partial recovery may have been due to the true loss of genes in the specific lineage or due to genome reduction, assembly fragmentation, or differences in gene annotation.

Genome clustering by iron and sulfur scores resulted in three distinct metabolic groups (Figure 3b). Genomes enriched in iron (*n* = 12) showed high scores in the iron-entry (*cyc2/rus/cyc1*), respiratory electron-transfer (*petABC/bc1*), and terminal oxidase (*coxABCD*) modules (IMS > 0.65, SMS < 0.45). Sulfur-enriched genomes (*n* = 9) were enriched in sulfur-oxidation and sulfur-transfer modules (SMS > 0.50, IMS < 0.55) with high representation of *Sox*, *SQR/TetH/Dox,* and *Hdr/Tus/DsrE* modules. Mixed genomes (*n* = 10) maintained a strong contribution of both pathway groups (IMS > 0.60, SMS > 0.50).

Eight representative genomes were selected for detailed pathway and locus-level analysis, representing the major metabolic profiles identified across the dataset (Table 2). Iron-enriched representatives (GCF_000021485.1, GCF_000214095.2) showed strong recovery of *rus*, *petABC*, *coxABCD,* and loci associated with cytochromes. Mixed representatives (GCF_016250455.1, GCF_046254925.1, GCF_900174455.1) kept iron and sulfur modules such as *rus*, *petABC*, *coxABCD*, *soxB*, *soxYZ*, *tetH*, *doxDA-like,* and *Hdr/Tus/DsrE* components. Sulfur-enriched representatives (GCF_030996345.1, GCF_018853465.2, GCF_047421495.1) had lower iron-associated recovery but strong representation of *soxABXYZ, sqr*, *tetH*, *doxX,* and *Hdr/Tus/DsrE* modules.

For each of the marker-gene recovery thresholds (40%, 50%, and 60%), recovery frequencies of module types were computed to assess their sensitivity to the different thresholds. The pet complex was isolated in 17 genomes at the 60% threshold, 19 genomes at the 40% threshold, and 17 genomes at the 50% threshold, while the iron-entry module was isolated in 21 genomes at all three thresholds. The recovery of the *SQR/TetH/Dox* module also remained the same, with 12 genomes meeting each of the thresholds. The modules that were more sensitive to the threshold applied were the terminal oxidase, *Sox*, and *Hdr/Tus/DsrE* modules. Only 21 genomes were recovered at the 40% and 50% terminal oxidase thresholds, but at 60%, only 10 genomes were recovered, and no genomes were recovered for Sox. At the 40% threshold, the *Hdr/Tus/DsrE* module was recovered in 29 genomes, and at the 50% and 60% thresholds, in 22 genomes. The results indicate that the main patterns remained stable in some modules, and that the others, more often represented by partial gene complements, were more sensitive to the more strict recovery criteria. All results are presented in the Supplementary Table S2 and Figure S1.

### 2.6. Conserved and Variable Pathway Families Supported by Orthogroup Analysis

Curated marker assignments were validated based on conserved-domain analysis and orthology inference for accurate functional annotation. Many candidate proteins carried domains consistent with cytochrome-mediated electron transfer (*cytochrome c-type heme-binding* domains, Rieske [2Fe-2S] domains), sulfur oxidation (*SoxX/Y/Z* domains, tetrathionate hydrolase domains), sulfur transfer (*Rhodanese-like* domains, *TusA sulfur transferase* domains), and general redox metabolism.

Annotations such as “*cytochrome*”, “*oxidoreductase*” or “*sulfur carrier protein*” were handled with caution in the absence of other evidence supporting their assignment. EggNOG annotations and orthogroup assignments were used to further separate pathway-relevant homologs from unrelated redox proteins. This was particularly true for proteins related to cytochromes, *Dox/TQO-like* proteins, and sulfur-transfer proteins, which are often inconsistently annotated between genomes.

Orthogroup inference for the 95,180 predicted proteins revealed conserved and variable protein families across the 31 genomes. Mapping of marker genes on orthogroups was used to check if the proteins in pathways belonged to consistent homologous families. Orthogroups with respiratory electron-transfer functions were generally larger, more widely distributed across genomes, and contained iron-associated markers. For example, the Rus orthogroup had 21 members in 21 genomes, and the *CoxA* orthogroup had 21 members, consistent with the detection frequencies of the modules. In contrast, sulfur-associated markers were more heterogeneous in their orthogroup composition, with variable membership. The *SoxB* orthogroup comprised 10 members, and the *TusA* orthogroup comprised 22 members, confirming the differential conservation of these systems.

The same pattern was observed in the gene-content matrix, where iron-related modules were generally more conserved than sulfur-related modules. The combined validation approach confirmed that observed pathway patterns represented biological variation, not annotation artifacts.

### 2.7. Gene Neighborhood Architecture Reveals Conserved and Fragmented Pathway Loci

**Figure 4:**
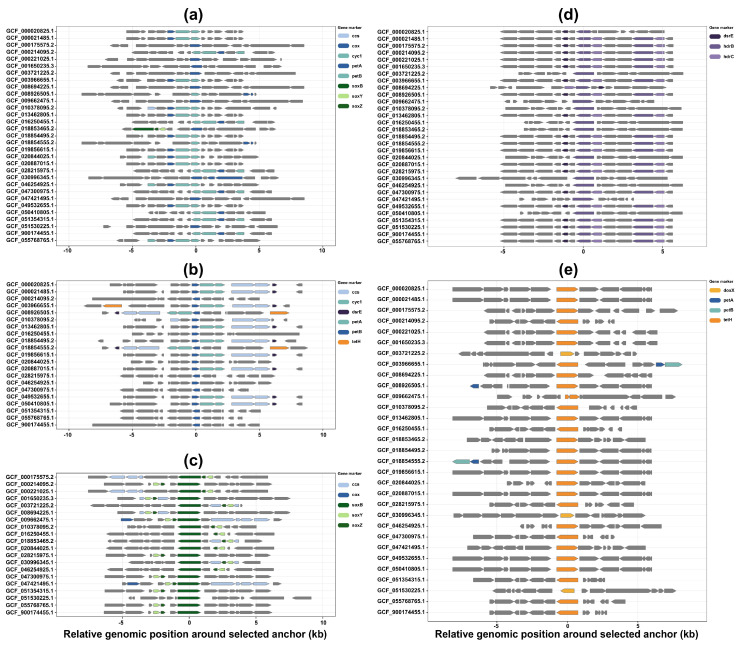
Gene-neighborhood architecture of representative iron- and sulfur-associated pathway loci in *Acidithiobacillus* genomes. Gene neighborhoods are shown relative to the selected anchor gene, with genomic coordinates expressed as relative distance (kb). Arrows indicate predicted coding sequences and their transcriptional orientation. Colored arrows represent curated pathway marker genes, whereas gray arrows represent neighboring genes. (**a**) *rus/cyc1/cox* iron-entry locus. (**b**) *petABC/bc1* respiratory electron-transfer locus. (**c**) Terminal oxidase-associated locus containing *coxABCD* and cytochrome maturation genes (*ccsA*, *ccsB*, *ctaG*). (**d**) Sox sulfur-oxidation locus (*soxABXYZ*). (**e**) *Hdr/Tus/DsrE* sulfur-transfer locus (*hdrBC*, *tusA*, *dsrE*-like).

The analysis of gene neighborhoods in eight representative genomes revealed that iron- and sulfur-associated markers are arranged in structured genomic regions, and not as isolated genes (Figure 4). The iron gene-neighborhood dataset contained 211 entries in eight genomes, grouped into 13 curated classes. Iron-associated loci included *rus/cyc1/cox regions* (5 entries), *petABC/bc1* electron transfer loci (11 entries), terminal oxidase loci (33 entries), and *cytochrome* support loci (30 entries). A conserved ~15–20 kb region was observed in iron-enriched genomes where *rus*, *cyc1,* and *cox* genes were co-localized. In sulfur-enriched genomes, arrangements were fragmented with genes scattered in distant genomic regions.

The dataset of sulfur gene neighborhoods comprised 146 entries clustered in 13 curated classes. Sulfur-associated loci included Sox operons (11 entries) and *SQR/TetH/Dox* regions and *Hdr/Tus/DsrE* sulfur-transfer loci (34 entries). In sulfur-enriched and mixed genomes, *Sox* loci were arranged in a conserved *soxA-soxB-soxX-soxY-soxZ* order, whereas Sox loci were not identified in iron-enriched genomes. The *Hdr/Tus/DsrE* loci were represented in all metabolic profiles with conserved clustering of *hdrBC*, *tusA,* and *dsrE-like* genes, suggesting sulfur-transfer capacity is a broadly retained core function.

Synteny analysis revealed a striking difference, with iron-associated systems having few types of loci that were conserved or missing, while sulfur-associated systems showed a greater diversity in their combinations of processing regions. This modular organization explains the higher diversity of sulfur-associated gene profiles and supports differential pathway retention as a driver of metabolic diversification in *Acidithiobacillus*.

### 2.8. Classification of Synteny: Conserved, Partial, Fragmented and Weak Loci

The pathway-associated neighborhoods in the eight representative genomes were classified by the degree of syntenic conservation. A locus was considered conserved if pathway markers were predicted to be present in the same genomic region, with similar gene order and orientation, across genomes. A subset of the expected markers or limited rearrangements were contained in partially conserved loci. Genes in fragmented or weak loci were distributed across distant genomic regions or broken up by assembly breaks.

With this classification, this study identified 145 pathway-associated loci, including 79 iron-associated and 66 sulfur-associated. Iron-associated loci were characterized by terminal oxidase and cytochrome-support sections; sulfur-associated loci were characterized by *Hdr/Tus/DsrE* sulfur-transfer neighborhoods and *Dox*-associated sulfur-processing regions (Table 3).

Synteny analysis suggests that the locus explains pathway conservation better than do individual marker genes. Conserved loci gave strong support for maintenance of functional pathways, while fragmented loci indicated pathway reduction, chromosomal rearrangement, or assembly disruption. The results highlight the importance of the genetic context for the reconstruction of metabolic pathways and confirm the modular character of iron and sulfur metabolism.

### 2.9. Phylogenomic Mapping Links Pathway Modules and Evolutionary Architecture

The functional relationship between the iron and sulfur modules was mapped onto the OrthoFinder species tree using the module-completeness profiles, and it was found that closely related genomes often had similar sets of iron and sulfur modules. The above observations are consistent with the notion that pathway organization is related to phylogenetic relatedness. There were exceptions as well, which may indicate that the genomic diversity observed was caused by gene loss, local genomic rearrangement, and differential pathway retention.

**Figure 5:**
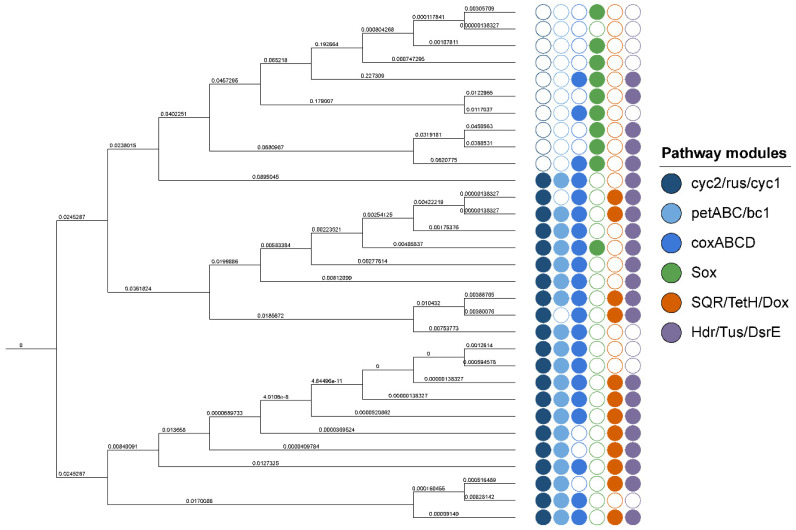
Phylogenomic distribution of curated iron- and sulfur-associated pathway modules across 31 *Acidithiobacillus* genomes. The OrthoFinder species tree is annotated with the recovery of six pathway modules (*cyc2/rus/cyc1*, *petABC/bc1*, *coxABCD*, *Sox*, *SQR/TetH/Dox*, and *Hdr/Tus/DsrE*). Each column represents one pathway module, and each row corresponds to a genome in the phylogeny. Filled circles indicate recovery of the corresponding module, whereas open circles indicate that the module was absent or incompletely recovered. Filled colored circles indicate recovery of the corresponding pathway module, whereas open (unfilled) circles with colored outlines indicate that the module was absent or incompletely recovered. Circle colors correspond to the pathway modules shown in the legend.

Iron-linked modules (*cyc2/rus/cyc1*, *petABC/bc1*, *coxABCD*) were distributed among various phylogenetic branches and were generally more conserved than sulfur-linked modules. In contrast, sulfur-related systems showed an increase in variability. *Sox* modules were found in a more limited phylogenetic distribution, while the *Hdr/Tus/DsrE sulfur*-transfer systems were found in several phylogenetic groups. The intermediate distribution of *SQR/TetH/Dox* modules further supported several sulfur metabolism strategies. The phylogenomic distribution of these curated pathway modules in Figure 5, placing the observed metabolic diversity into an evolutionary context.

### 2.10. An Integrated Model of Pathway Diversity in Acidithiobacillus

An integrated approach combining gene content analysis, module scoring, validation of conserved domains, orthogroup inference, gene-neighborhood reconstruction, synteny classification, and phylogenomic mapping supports a unified model of pathway diversification. No conserved chemolithotrophic pathway was identified, but different metabolic configurations are defined by different combinations of iron- and sulfur-associated modules.

Three major genomic configurations were identified:

1. Iron-enriched genomes retained robust complements of *cyc2/rus/cyc1*, *petABC/bc1*, and *coxABCD* modules, with the greatest predicted potential for ferrous iron oxidation associated with aerobic respiration.
2. Sulfur-enriched genomes showed increased representation of *Sox*, *SQR/TetH/Dox*, and *Hdr/Tus/DsrE* modules and less conservation of iron-entry systems, suggesting increased specialization for sulfur oxidation and trafficking.
3. Mixed genomes may possess the greatest predicted metabolic flexibility, retaining high numbers of both iron- and sulfur-associated modules, with conserved respiratory electron-transfer systems and multiple sulfur-processing pathways.

**Figure 6:**
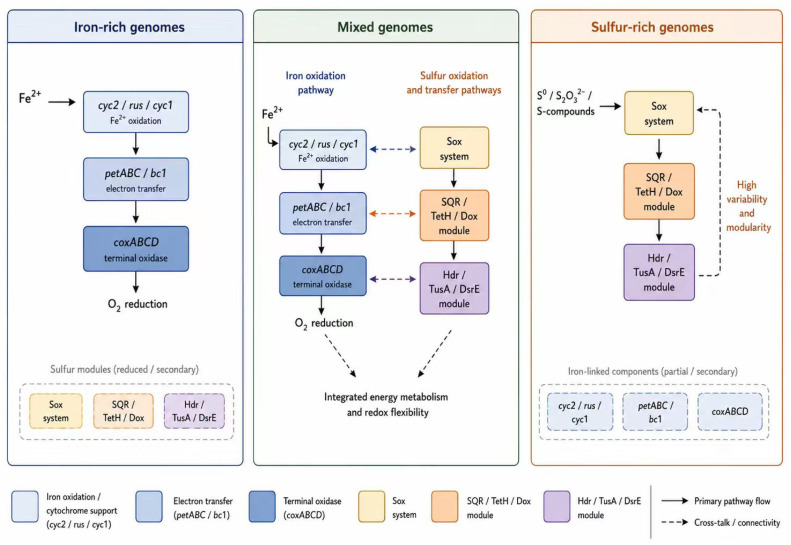
Integrated model of the diversification of iron- and sulfur-oxidation pathways in Acidithiobacillus. The model represents three main genomic patterns found in this study: iron-enriched, sulfur-enriched and mixed iron-sulfur. Solid black arrows represent flow of primary pathway; dashed arrows represent inferred functional interactions or connectivity between pathway modules. Putative relationships between iron and sulfur modules are shown by dashed blue and orange arrows, and black dashed arrows show convergence to form integrated energy metabolism pathways. The findings indicate that respiratory modules containing iron and the sulfur-processing systems have been diversified throughout the genus via conserved elements, which are particularly important for the diversification of the respiratory pathways. The results suggest that the diversification of respiratory pathways throughout the genus was driven by the conserved respiratory modules containing iron, and sulfur-processing systems.

The integrated model also shows that sulfur metabolism is evolving more dynamically than iron metabolism. Iron-associated respiratory modules were relatively conserved and frequently associated with structured genomic loci. Sulfur-associated pathways were more variable in gene content, neighborhood organization, and phylogenomic distribution. This pattern is consistent with differential conservation and diversification of sulfur-oxidation systems across the genus. This observation leads us to propose a modular retention model of the evolution of *Acidithiobacillus* energy metabolism with pathway reduction and reorganization at the local level, leading to iron-enriched energy metabolism, sulfur-enriched energy metabolism, and mixed energy metabolisms, which may represent an ecologic adaptation to the acidic environment with a high concentration of sulfurous minerals (Figure 6).

## 3. Discussion

### 3.1. Modular Genomic Organization of Iron Oxidation

The iron-associated modules were among the most conserved metabolic systems found in this study. The *cyc2/rus/cyc1* iron-entry module and *coxABCD* terminal oxidase complex were identified in 67.7% of genomes, and the *petABC/bc1* respiratory electron-transfer module was identified in 61.3%. The frequent co-occurrence and conserved genomic organization of these modules are in agreement with the model of iron oxidation as an integrated electron-transfer chain rather than a collection of independent genes [1,38]. This architecture, in which outer-membrane iron oxidation *(cyc2*), periplasmic electron transfer (*rus*, *cyc1*), respiratory electron transfer (*petABC/bc1*), and terminal oxygen reduction (*coxABCD*) are coordinated via structured genomic loci, likely allows for efficient energy conservation from ferrous iron oxidation [19,39,40].

The absence of complete iron modules in about one-third of genomes suggests that ferrous iron oxidation is not a ubiquitous feature of the genus. This variation may reflect lineage-specific adaptation to environments where iron is not the primary energy source or where alternative electron acceptors are available [38]. Interestingly, several genomes missing the *cyc2/rus/cyc1* module still retained *petABC/bc1* and *coxABCD* components, implying that respiratory electron-transfer functions might have roles beyond iron oxidation, possibly in sulfur-based energy conservation or redox balance maintenance [39]. Synteny analysis also showed that iron-associated genes are organized around a limited number of conserved locus types that are either retained as intact systems or lost, supporting the interpretation that iron oxidation capacity is maintained or lost as a functional unit.

### 3.2. Diversity and Flexibility of Sulfur Metabolism

Compared with iron-associated modules, sulfur-associated modules exhibited broader differences in gene content and module recovery across the analyzed genomes. The *Sox* module was limited to 32.3% of genomes, the *SQR/TetH/Dox* module was found in 38.7%, and the *Hdr/Tus/DsrE* sulfur-transfer module was the most widely conserved at 71.0%. The uneven distribution confirms that sulfur oxidation in *Acidithiobacillus* is not governed by a single conserved pathway but by multiple, partially overlapping systems [41].

The limited distribution of *Sox* genes indicates that thiosulfate oxidation mediated by Sox is a lineage-specific strategy and not a core function. Several genomes without *Sox* genes still contained *Hdr/Tus/DsrE* or *SQR/TetH/Dox* modules, indicating that sulfur metabolism can be maintained via alternative pathways. This is in agreement with previous suggestions that sulfur oxidation pathways in acidophiles are highly flexible and substrate-dependent [42,43]. The detection of *SQR/TetH/Dox* modules in *Sox*-positive and *Sox*-negative genomes also provides evidence that multiple strategies for processing sulfur can co-occur or be used interchangeably.

The conserved genomic organization and broad distribution of the *Hdr/Tus/DsrE* module may represent a core sulfur-trafficking function. The *Hdr/Tus/DsrE* module is thought to play a major role in intracellular sulfur transfer and processing, unlike the *Sox* and *SQR/TetH/Dox* systems, which are directly coupled to sulfur oxidation reactions. Therefore, the fact that it is widely conserved should not be taken as evidence that it represents a stand-alone sulfur oxidation pathway. The consistent co-localization of *hdrBC*, tusA and *dsrE-like* genes in conserved genomic regions suggests that these systems may play an important role in intracellular sulfur transfer and sulfur intermediate processing [41]. This is in line with recent studies that have emphasized the importance of *Hdr-like* complexes and sulfur-carrying proteins in sulfur oxidation networks [19]. The greater conservation of this module than *Sox* supports the hypothesis that sulfur transfer processes are more evolutionarily stable than specific sulfur oxidation pathways.

### 3.3. Evolutionary Implications and Genomic Configurations

Phylogenomic mapping showed that module distributions follow lineage-associated patterns, such that closely related genomes tend to share similar metabolic profiles, indicating that vertical inheritance is important for pathway conservation [38]. However, exceptions such as variable *Sox* retention in closely related *A. caldus* strains suggest that metabolic diversification has also been driven by gene loss, gain, and rearrangement.

Three major genomic configurations were identified: iron-enriched (n = 12), sulfur-enriched (n = 9), and mixed (n = 10). Genomes enriched in iron, dominated by lineages of A. ferrooxidans, contain intact systems for iron entry, respiratory electron-transfer, and terminal oxidase, suggesting the potential for specialization in ferrous iron oxidation coupled with aerobic respiration. The sulfur-enriched genomes, mainly represented by *A. thiooxidans* and *A. caldus* lineages, display reduced iron modules but high abundance of sulfur-linked systems, consistent with specialization for sulfur-based energy acquisition. Mixed genomes, present in several lineages, preserve large portions of both iron and sulfur systems and may possess greater metabolic flexibility.

These results have important implications for the application of bioleaching in industry. Iron-enriched genomes may possess greater potential for ferric iron regeneration, an important step in sulfide mineral dissolution [43]. Sulfur-enriched genomes may be better adapted to sulfur turnover and removal of inhibitory sulfur intermediates that can reduce mineral accessibility and leaching efficiency [3,13]. Mixed genomes that still have both systems could be the most versatile for bioleaching operations where both types of substrates are present [5,44]. Genomic profiling may provide a useful framework for selecting strains for biomining applications based on their complete module architectures and not on individual marker genes.

### 3.4. Limitations and Future Direction

Several limitations should be mentioned in interpreting these results. First, genomic analyses predict metabolic potential and not physiological activity; the presence of complete pathway modules does not necessarily imply gene expression or pathway function under certain environmental conditions [28]. Secondly, three of the 31 genomes analyzed were draft assemblies, which may bias estimates of pathway completeness and synteny interpretation, as missing genes may be due to assembly fragmentation and not true biological absence. However, these draft assemblies represent only a small percentage of the dataset (3/31 genomes) and the main conclusions of this study are based on comparative patterns across the whole dataset and not on individual genomes. Therefore, classifications of fragmented loci and partially recovered modules in these draft genomes should be taken with caution, although they are unlikely to greatly affect the overall comparative trends identified in this study.

Third, the module-scoring framework (≥50% detection threshold) oversimplifies complex biological systems and may fail to capture all functionally relevant variation, as some genes within a module may be essential while others are dispensable [13]. Fourth, although we performed multi-layer validation of annotations using conserved-domain analysis, eggNOG annotations, and manual curation, the annotation and orthology inference are not perfect, particularly for proteins in large families with broad functional annotations, such as *cytochromes* and *oxidoreductases* [15,45]. Finally, the module-based classifications (iron-enriched, sulfur-enriched, mixed) are comparative genomic categories and should not be interpreted as direct measures of physiological activity or bioleaching efficiency. Future investigations based on transcriptomics, proteomics, and metabolomics combined with physiological assays are required to verify these genomic predictions [46].

A sensitivity analysis using module recovery thresholds of 40%, 50%, and 60% also revealed that the impact of the different thresholds was related to the different functional modules. The iron-entry and the *SQR/TetH/Dox* modules did not change across all the thresholds evaluated, and there was only a small decrease at the most stringent value for the pet complex. The *Sox (terminal oxidase)* and *Hdr/Tus/DsrE (Hdr)* modules, in contrast, showed a greater response to the increasing thresholds, meaning that several genomes only had a partial complement of their marker genes. From these observations, certain pathways are relatively conserved while others have a greater degree of pathway-specific evolutionary plasticity and flexibility: gene loss, pathway fragmentation, or functional substitution by lineage. This resulted in the selection of the 50% criterion as an intermediate operational criterion that minimizes the risk of over-interpretation when it comes to gene detections that occur in isolation, and does not miss too many partially conserved modules. However, the changes relating to the recovery of a module should be taken in conjunction with the genomic context and distribution of the gene encoding the marker in the module and not as proof of physiological activity.

## 4. Materials and Methods

### 4.1. Genome Dataset Collection and Quality Assessment

Genus assemblies of the *Acidithiobacillus* genus were retrieved from the NCBI Genome database. A total of 31 publicly available genomes were selected to represent phylogenetic and strain-level diversity within the genus, with preference given to complete or high-quality assemblies. The dataset included representatives of *A. ferrooxidans* (n = 8), *A. thiooxidans* (n = 7), A. caldus (n = 6), *A. ferrivorans* (n = 4), *A. ferridurans* (n = 3) and three other *Acidithiobacillus* genomes from other species in the genus. For each genome, accession number, species assignment, strain designation, assembly level, genome size, GC content, completeness, contamination, number of predicted coding sequences and assembly statistics were recorded. Transparency and reproducibility of the comparative dataset are achieved by providing the full list of genomes and metadata in Supplementary Table S1. Genome quality was assessed before downstream analysis to minimize the influence of incomplete and contaminated assemblies [16].

Q=C−5T

where Q = genome quality score, C = completeness, and T = contamination. Contamination was assigned a greater penalty because contaminating sequences might artificially increase gene presence in comparative analyses. Genome-quality interpretation was performed based on principles of microbial genome assessment tools such as CheckM2 [47] and assembly assessment methods like QUAST [48].

### 4.2. Genome Annotation and Functional Gene Identification

All genomes were re-annotated using Bakta v1.9.3 [29] and Prokka v1.14.6 [49] with the default parameters to minimize annotation variation between sources. Protein-coding sequences were predicted and functionally annotated based on gene names, product descriptions, conserved domains, and similarity to reference proteins [9]. The annotation process leveraged several resources: eggNOG-mapper v2 [45,50] for orthology-based functional assignment, InterProScan v5.57-90.0 [35] for conserved-domain identification, and KofamKOALA [36] for KEGG Orthology assignment.

Genes were identified relative to markers for ferrous iron oxidation, reduced sulfur compound oxidation, electron transfer, and terminal oxidase activity. The iron oxidation gene set was comprised of *rus*, *cyc1*, *cyc2-like*, *coxA-D,* and *petA-F* [44]. The sulfur oxidation gene set included *soxA-D*, *sqr*, *tetH*, *doxA*, *doxD*, *hdrA-C*, *tusA,* and *dsrE-like* genes. Only candidate genes for which annotation, conserved-domain evidence, orthology assignment, and sequence similarity consistently supported the same function were accepted [10]. Genes were considered present if BLASTP (v2.17.0+ (NCBI BLAST+)) revealed ≥40% identity and ≥70% coverage against reference sequences. To avoid overestimation of pathway completeness, genes with generic descriptions, low similarity, or ambiguous annotation were filtered out [51,52].

Orthology assignments from eggNOG-mapper and OrthoFinder were used in functional annotation but were not used as the sole criterion for gene identification. However, no similarities were found in the functions of the members of the same orthogroup because orthogroups might also contain paralogous proteins with different functions. The candidate proteins were scored individually by a combination of orthogroup, conserved-domain architecture, similarity to proteins with known structures, and neighborhood in the genome. Manual curation of proteins was done within the set of proteins containing general annotations like ‘*cytochrome*’ or ‘*oxidoreductase*’ or ‘*sulfur carrier protein*’, and functional assignments were accepted only if they agreed with other independent lines of evidence. Proteins that had limited evidence and were ambiguously annotated, had ambiguous domains, or had conflicting annotations and domains were not included in the curated protein marker set.

### 4.3. Presence–Absence Matrix Construction

A binary matrix was built to compare the distribution of iron and sulfur oxidation genes among the 31 genomes. Each gene was scored as present or absent in each genome according to the following rule:

$$G\in \left\{0\;1\right\}n\times m$$

where n=31 genomes and m= the total number of curated marker genes. A value of 1 indicates that the gene was detected and a value of 0 indicates that it was not detected. This matrix was the main dataset used to compare the distribution of metabolic genes in the genus. Similar presence–absence approaches are widely used in comparative microbial genomics and orthology-based genome studies, e.g., the OrthoFinder framework [53].

### 4.4. Gene Conservation and Distribution Analysis

To identify conserved and variable pathway components, the frequency of each gene was calculated:

$$F_{j}=\frac{\sum_{i-1}^{n}G_{ij}}{n}\times 100$$

where F_j_ is the frequency of gene j, G_ij_ is the binary presence of gene j in genome i, and n is the total number of genomes. High F_j_ values indicate widespread distribution; low values indicate restricted distribution. Genes shared by most genomes were treated as conserved components, and those with limited distribution as variable components reflecting strain-level adaptation, gene loss, or pathway specialization [19].

### 4.5. Metabolic Gene Profiles Classification

Genomes were classified according to the relative proportion of genes for iron oxidation and sulfur oxidation. Total counts were determined separately:

$$I_{i}={\Sigma_{j-1}^{m}G}_{ij}^{iron}\;s_{i}=\Sigma_{j-1}^{k}G_{ij}^{Sulfur}$$

where I_i_ is the number of iron oxidation genes, S_i_ is the number of sulfur oxidation genes, m is the number of iron-related genes, and k is the number of sulfur-related genes. Iron-rich genomes were enriched in genes for iron oxidation, sulfur-rich genomes were enriched in sulfur oxidation profiles, and mixed genomes contained both dominant components. These classifications were used as comparative genomic categories rather than as physiological measures. The iron-entry module is specifically used throughout this work to refer to the combination of *cyc2/rus/cyc1* genes involved in ferrous iron oxidation and electron transfer from iron to the respiratory chain. In contrast, the iron oxidation pathway is used in a broader sense to refer to the entire iron-dependent energy-conserving pathway, including the iron-entry (*cyc2/rus/cyc1*) and electron transfer to *coxABCD* modules. Thus, these terms are not used as synonyms.

### 4.6. Iron and Sulfur Module Scoring

In order to allow a more precise assessment of pathway completeness, genes were grouped into functional modules. The primary components involved in iron oxidation were the *rus/cyc* electron transfer module, the cox terminal oxidase module, and the pet *cytochrome bc1* module [10]. The score of the iron module was calculated as follows:

IMSi=Ri/3+Ci/3+Pi/3

where (IMS_i_) denotes the iron module score for genome (i), (R_i_) denotes the rus/cyc module score, (C_i_) denotes the cox module score, and (P_i_) denotes the pet module score.

The major modules for sulfur oxidation were the Sox system, the *sqr/tetH/dox* group, and the *hdr/tusA/dsrE-like* group. The sulfur module score was calculated as follows:

SMSi=SOXi/3+STDi/3+HTDi/3

where (SMS_i_) denotes the score of the sulfur module for genome (i), (SOX_i_) denotes the score of the *Sox* module, (STD_i_) denotes the score of the *sqr/tetH/dox* module, and (HTD_i_) denotes the score of the hdr/tusA/dsrE-like module.

A value close to 1 indicates that most of the genes in the module were detected, whereas a value close to 0 indicates that the module was poorly represented or absent. These scores were comparative measures of genome potential [25]. They were not regarded as direct evidence of gene expression, enzyme activity, or bioleaching rate because physiological activity requires experimental confirmation by transcriptomic, proteomic, biochemical, or culture-based analysis. For the interpretation of sulfur and iron metabolism, this difference is relevant [25].

The criterion for module recovery was evaluated by applying three recovery threshold levels (40%, 50%, and 60% of the expected marker genes in a module). The proportion of marker genes detected in each genome relative to the total number of marker genes corresponding to that module was calculated. The recovery of the module was acknowledged when the ratio of the detected marker genes was equal to or higher than the established threshold. Recovery rates were computed separately for each of the modules: iron-entry module, pet complex module, terminal oxidase module, *Sox* module, *SQR/TetH/Dox* module, and *Hdr/Tus/DsrE* module. The threshold of 50% was utilized as the main criterion since it represents an intermediate and, thus, biologically reasonable level of completion of the module. The conducted sensitivity analysis confirmed that a greater probability of module recovery was observed for each of the applied assessment thresholds. The performed sensitivity analysis was only meant to demonstrate the module detection properties and was not aimed at redefining the classifications of mixed, iron- and sulfur-enriched genomes based on IMS and SMS classification scores.

### 4.7. Phylogenomic Analysis

Phylogenomic relationships were reconstructed using conserved single-copy orthologous genes found across the dataset. Alignments were performed on orthologous proteins to infer genome-level relationships, giving higher resolution than single-gene analysis [54]. Model-based phylogenetic methods were used to reconstruct a maximum-likelihood phylogenetic tree. Model selection and tree inference were performed using methods implemented in IQ-TREE 2 [48] and Model Finder [49]. 16S rRNA sequences were used as a secondary taxonomic comparison, but not as the main evolutionary framework. The obtained tree was compared with the gene matrix to assess whether metabolic profiles were in accordance with species-level relationships, or whether strain-specific variability was present [19].

### 4.8. Operon Organization and Gene Neighborhood

Representative genomes were selected for gene neighborhood analysis based on phylogenomic position, species coverage, and variation in iron and sulfur gene content. The analysis compared genomic regions containing target genes for gene order, gene orientation, gene clustering, and neighboring genes [38]. Iron oxidation regions contained genes such as *rus*, *cyc1*, *cyc2-like*, *cox*, and *pet*, while sulfur oxidation regions contained genes such as *sox*, *sqr*, *tetH*, *dox*, *hdr*, *tusA*, and *dsrE-like* [7].

The following score was used for estimating synteny conservation:

$$ss=\frac{N_{C}}{N_{t}}$$

where SS is the synteny score, N꜀ is the number of conserved neighboring gene pairs, and N_t_ is the total number of neighboring gene pairs compared. A higher score indicates better conservation of gene order.

### 4.9. Gene Co-Occurrence Analysis

Genes were analyzed for co-occurrence to identify those that co-occur across genomes. The pairwise co-occurrence between two genes was calculated as follows:

$$CO_{ab}=\frac{\sum_{i-1}^{n}G_{ia}G_{ib}}{n}$$

where (CO_ab_) denotes the co-occurrence score between genes (a) and (b), (G_ia_) and (G_ib_) denote their presence in genome (i), and (n) denotes the total number of genomes. A high co-occurrence score indicates two genes were detected together repeatedly. This analysis was utilized to help with the interpretation of iron and sulfur modules, specifically gene groups such as *rus/cyc*, *cox*, *pet*, *sox*, *sqr/tetH/dox,* and *hdr/tusA/dsrE-like*. Co-occurrence was interpreted with caution, as genes could be together due to a common pathway function, physical linkage, common ancestry, or similar environmental selection.

### 4.10. Principal Component Analysis and Cluster Analysis

Principal component analysis on the binary gene matrix was performed to assess whether the genomes clustered according to their iron and sulfur oxidation gene profiles. Constant genes do not contribute to separation among genomes, so genes with no variation across the dataset were removed before analysis. The general transformation of PCA was given as follows:

PC=XW

where (PC) denotes the principal component scores, (X) denotes the centered gene matrix, and (W) denotes the component loadings. In this analysis, PCA was used to reduce the main differences in gene composition among genomes and to explore whether iron-rich, sulfur-rich, and mixed groups exhibited separation.

Hierarchical clustering was also used to compare the similarity of genomes based on binary gene profiles. The general distance between two genomes was given as follows:

$$D_{ab}=1-S_{ab}$$

where (D_ab_) denotes the distance between genomes (a) and (b), and (S_ab_) denotes the similarity of the genomes based on shared gene presence. Genomes with similar iron and sulfur oxidation profiles clustered closer. These analyses were used to complement biological interpretation rather than to replace phylogenomic analysis.

### 4.11. Comparative Interpretation Integration

The final interpretation combined gene presence–absence patterns and frequencies, module scores, phylogenomic placement, co-occurrence patterns, and gene neighborhood organization. Conserved genes were considered as stable elements of energy metabolism at the genus level, whereas variable genes and rearranged loci were indicative of adaptation, pathway diversification, or strain-specific evolution [51]. This is consistent with studies showing that acidophilic chemolithotrophs have conserved core features but variable gene content associated with sulfur oxidation, iron oxidation, plasmids, and environmental adaptation [28].

## 5. Conclusions

Comparative genomic analysis of 31 *Acidithiobacillus* genomes shows that the iron- and sulfur-oxidation systems have modular genomic architectures rather than being universally conserved pathways. Modules associated with iron (*cyc2/rus/cyc1*, *petABC/bc1*, *coxABCD*) are relatively conserved (61–68% of genomes), whereas the sulfur-associated system displays greater variation in gene content and module recovery, with *Sox* genes present in only 32% of genomes and *Hdr/Tus/DsrE* sulfur-transfer modules highly conserved in 71%. Three main genomic configurations (iron-enriched (n = 12), sulfur-enriched (n = 9) and mixed (n = 10)) indicate that metabolic diversification has been driven by differential module retention, loss and reorganization. Iron-enriched genomes dominated by *A. ferrooxidans* suggest they may be adapted for ferrous iron oxidation, while sulfur-enriched genomes dominated by *A. thiooxidans* and *A. caldus* suggest they may be adapted for sulfur-based energy acquisition. Mixed genomes retain substantial complements of both iron- and sulfur-associated modules and may possess greater metabolic flexibility. These results show that metabolic potential cannot be reliably predicted from single marker genes, but its prediction requires the integration of orthology, genomic context, and phylogenomic relationships. These results provide a comparative genomic framework for identifying strains with potential for bioleaching and ecological function in *Acidithiobacillus*. However, these predictions are based on genomic evidence and require validation through physiological experiments and integrative approaches combining transcriptomics, proteomics, and metabolomics to determine their ecological performance and bioleaching efficiency in future studies.

## Figures and Tables

**Table 1:** Characteristics of the eight representative *Acidithiobacillus* genomes selected for detailed gene neighborhood and synteny analyses.

Module Class	Functional Module	Main Marker Genes Included	Genomes with Module Detected	Frequency Among 31 Genomes	Interpretation
Iron oxidation	Iron-entry/periplasmic electron-transfer module	*cyc2-like*, *rus*, *cyc1*	21	67.7%	Frequent but not universal iron-entry system
Respiratory electron transfer	*petABC/bc1* module	*petA*, *petB*, *petC* and related *bc1 components*	19	61.3%	Respiratory electron-transfer complex widely conserved
Terminal oxidation	Terminal oxidase module	*coxA*, *coxB*, *coxC*, *coxD*	21	67.7%	Respiratory electron transfer module-widely conserved
Sulfur oxidation	*Sox* module	*soxA*, *soxB*, *soxC*, *soxD* and related *Sox markers*	10	32.3%	Restricted sulfur-oxidation module found in a subset of genomes
Sulfur oxidation	*SQR/TetH/Dox* module	*sqr*, *tetH*, *doxA*, *doxD*, *doxDA-like*, *doxX*	12	38.7%	Intermediate-frequency sulfur module linked to sulfide, *tetrathionate* and *Dox/TQO-like* reactions
Sulfur transfer	*Hdr/Tus/DsrE* module	*hdrA*, *hdrB*, *hdrC*, *tusA*, *dsrE-like*	22	71.0%	Most conserved sulfur-related module kept, suggesting conserved sulfur transfer capacity

These genomes were chosen to represent iron-rich, sulfur-rich, and mixed metabolic profiles identified across the complete dataset. The complete list of all 31 genomes included in the comparative genomic analysis, together with accession numbers, species assignments, strain information, and genome quality metrics, is provided in Supplementary Table S1.

**Table 2:** Eight representative *Acidithiobacillus* genomes selected from the 31-genome dataset for detailed pathway, gene-neighborhood, and synteny analyses.

Accession Number	Genome Group	Iron Score	Sulfur Score	Dominant Iron-Associated Modules	Dominant Sulfur-Associated Modules	Pathway Interpretation
GCF_000021485.1	Iron-enriched	0.769	0.321	*rus*, *cyc1*, *petABC, coxABCD*, *cytochrome-associated loci*	*tetH*, *doxDA-like*, *doxX*, *hdrBC*, *tusA*, *dsrE-like*	Strong iron-linked respiratory profile with limited sulfur-processing capacity
GCF_000214095.2	Iron-enriched	0.769	0.541	*rus*, *petABC*, *coxABCD*, *cytochrome-associated loci*	*soxB*, *soxYZ*, *tetH*, *doxX*, *hdrBC*, *tusA*, *dsrE-like*	Iron-enriched genome with additional sulfur-associated pathways
GCF_016250455.1	Mixed	0.769	0.615	*rus*, *petABC*, *coxABCD*, *cytochrome-associated loci*	*soxB*, *soxYZ*, *tetH*, *doxDA-like*, *doxX*, *hdrBC*, *tusA*, *dsrE-like*	Broad mixed profile retaining both iron and sulfur modules
GCF_046254925.1	Mixed	0.769	0.615	*rus*, *petABC*, *coxABCD*, *cytochrome-associated loci*	*soxB*, *soxYZ*, *tetH*, *doxDA-like*, *doxX*, *hdrBC*, *tusA*, *dsrE-like*	Mixed metabolic profile with strong recovery of both pathway classes
GCF_900174455.1	Mixed	0.692	0.615	*rus*, *petABC*, *coxABCD*, *cytochrome-associated loci*	*soxB*, *soxYZ*, *tetH*, *doxDA-like*, *doxX*, *hdrBC*, *tusA*, *dsrE-like*	Mixed profile combining conserved iron and sulfur modules
GCF_030996345.1	Sulfur-enriched	0.321	0.538	*coxABCD and limited iron-associated loci*	*soxABXYZ*, *sqr*, *doxX*, *hdrBC*, *tusA*, *dsrE-like*	Sulfur-enriched genome with reduced iron-linked respiratory recovery
GCF_018853465.2	Sulfur-enriched	0.541	0.538	*coxABCD and limited iron-associated loci*	*soxABXYZ*, *tetH*, *doxX*, *hdrBC*, *tusA*, *dsrE-like*	Sulfur-enriched profile with strong Sox and sulfur-transfer recovery
GCF_047421495.1	Sulfur-enriched	0.541	0.538	*coxABCD and limited iron-associated loci*	*soxABXYZ*, *tetH*, *doxX*, *hdrBC*, *tusA*, *dsrE-like*	Sulfur-enriched profile dominated by sulfur oxidation and sulfur transfer

The iron and sulfur scores are normalized values derived from the presence–absence matrix and do not reflect physiological activity or gene expression levels.

**Table 3:** Locus-level organization of iron- and sulfur-associated modules in selected Acidithiobacillus genomes.

Pathway Class	Synteny/Locus Group	Loci/Features Detected	Main Genes or Modules Represented	Biological Interpretation
Iron-associated	Terminal oxidase locus	33	*coxA*, *coxB*, *coxC*, *coxD*	Most frequent iron-associated locus class supporting aerobic respiration
Iron-associated	*Cytochrome*-support locus	30	*Cytochrome c-associated genes*, *ccsA*, *ccsB*, *ctaG*	Supports cytochrome maturation and electron-transfer functions
Iron-associated	*petABC/bc1* electron-transfer locus	11	*petA*, *petB*, *petC*	Conserved respiratory electron-transfer module
Iron-associated	*rus/cyc1/cox* iron-associated locus	5	*rus*, *cyc1*, *cox genes*	Strong evidence for iron-linked electron-transfer architecture
Sulfur-associated	*Hdr/Tus/DsrE* sulfur-transfer locus	34	*hdrB*, *hdrC*, *tusA*, *dsrE-like*	Most frequent sulfur-associated locus supporting sulfur trafficking
Sulfur-associated	*Dox*/*thiosulfate*-associated locus	13	*doxDA-like*, *doxX*	Alternative sulfur-processing pathway
Sulfur-associated	*Sox* sulfur-oxidation locus	11	*soxA*, *soxB*, *soxX*, *soxY*, *soxZ*	Conserved sulfur-oxidation architecture in selected genomes
Sulfur-associated	*TetH tetrathionate* locus	7	*tetH*	Tetrathionate-associated sulfur oxidation
Sulfur-associated	*SQR* sulfide oxidation locus	1	*sqr*	Limited representation of sulfide oxidation pathway

## Data Availability

The data presented in this study are available upon request from the corresponding author.

## Supplementary Materials

The following supporting information can be downloaded at: https://www.mdpi.com/article/10.3390/ijms27156728/s1.
