# Supplementary material for: Comparative Genomic Analysis Reveals Distribution, Organization, and Evolution of Ferrous and Sulfur Oxidation Genes in the Genus Acidithiobacillus
Source: Int J Mol Sci. 2026 Jul 28;27(15):6728. doi: 10.3390/ijms27156728 (PMC13466987; doi:10.3390/ijms27156728)
Supplement: Supplementary file 1 [file ijms-27-06728-s001.zip › Figure S1 .pdf]

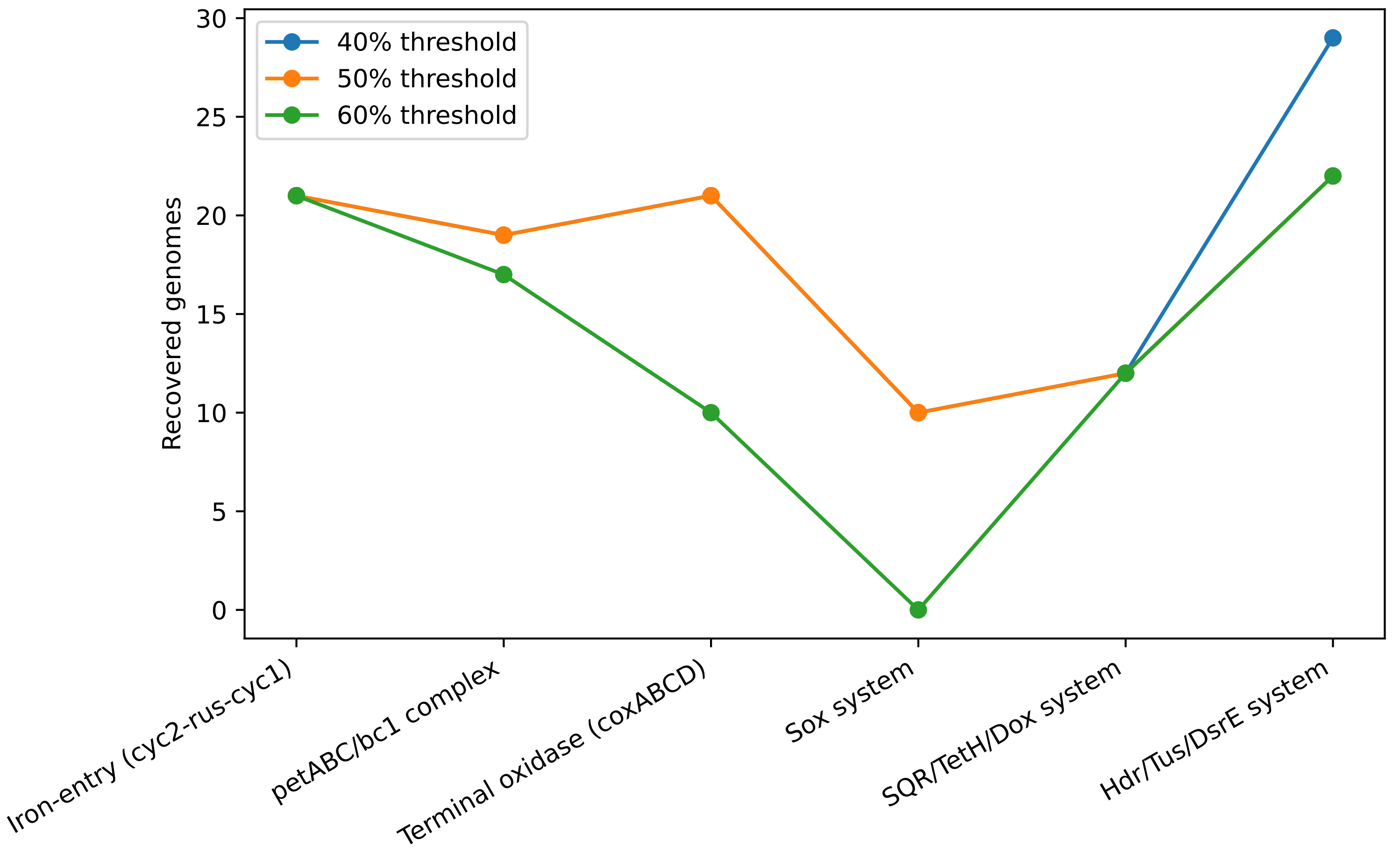
40% threshold
50% threshold
60% threshold
Recovered genomes
0
5
10
15
20
25
30
Iron-entry (cyc2-rus-cyc1)
petABC/bc1 complex
Terminal oxidase (coxABCD)
Sox system
SQR/TetH/Dox system
Hdr/Tus/DsrE system
